# Harmonized clinical trial methodologies for localized cutaneous leishmaniasis and potential for extensive network with capacities for clinical evaluation

**DOI:** 10.1371/journal.pntd.0006141

**Published:** 2018-01-12

**Authors:** Piero Olliaro, Max Grogl, Marina Boni, Edgar M. Carvalho, Houda Chebli, Mamoudou Cisse, Ermias Diro, Gláucia Fernandes Cota, Astrid C. Erber, Endalamaw Gadisa, Farhad Handjani, Ali Khamesipour, Alejandro Llanos-Cuentas, Liliana López Carvajal, Lise Grout, Badre Eddine Lmimouni, Mourad Mokni, Mohammad Sami Nahzat, Afif Ben Salah, Yusuf Ozbel, Juan Miguel Pascale, Nidia Rizzo Molina, Joelle Rode, Gustavo Romero, José Antonio Ruiz-Postigo, Nancy Gore Saravia, Jaime Soto, Soner Uzun, Vahid Mashayekhi, Ivan Dario Vélez, Florian Vogt, Olga Zerpa, Byron Arana

**Affiliations:** 1 Special Programme for Research and Training in Tropical Diseases (TDR), World Health Organization (WHO), Geneva, Switzerland; 2 Centre for Tropical Medicine and Vaccinology, Nuffield Department of Medicine, University of Oxford, Churchill Hospital, Oxford, United Kingdom; 3 U.S. Naval Medical Unit No.6 Peru, Lima, Peru; 4 Drugs for Neglected Diseases initiative (DNDi) Latin America, Rio de Janeiro, Brazil; 5 Fundação Oswaldo Cruz (Fiocruz), Salvador Bahia, Brazil; 6 Direction de l’Épidémiologie et de Lutte contre les Maladies, Division des Maladies Transmissibles, Service des Maladies Parasitaires, Rabat, Morocco; 7 Université Polytechnique de Bobo-Dioulasso, Bobo-Dioulasso, Burkina Faso; 8 Department of Internal Medicine, University of Gondar, Ethiopia; 9 Centro de Pesquisas René Rachou - Fundação Oswaldo Cruz – Minas, Minas Gerais, Brazil; 10 Centre for Tropical Medicine and Global Health, Nuffield Department of Medicine, University of Oxford, Oxford, United Kingdom; 11 Armauer Hansen Research Institute (AHRI), Addis Ababa, Ethiopia; 12 Molecular Dermatology Research Center, Faghihi Hospital, Shiraz University of Medical Sciences, Shiraz, Iran; 13 Center for Research and Training in Skin Diseases and Leprosy, Tehran University of Medical Sciences, Tehran, Iran; 14 Instituto de Medicina Tropical Alexander von Humboldt, Universidad Peruana Cayetano Heredia, Lima, Peru; 15 Programa de Estudio y Control de Enfermedades Tropicales (PECET), Universidad de Antioquia, Medellin, Colombia; 16 Neglected Tropical Diseases, World Health Organization, Geneva, Switzerland; 17 School of Medicine and Pharmacy, University Mohammed the fifth, Rabat, Morocco; 18 Dermatology Department, La Rabta Hospital, Tunis, Tunisia; 19 National Malaria & Leishmaniasis Control Program (NMLCP), Kabul, Afghanistan; 20 Service d'épidémiologie médicale, Institut Pasteur de Tunis, Tunis, Tunisia; 21 Department of Parasitology, Faculty of Medicine, Ege University, Izmir, Turkey; 22 Instituto Conmemorativo Gorgas de Estudios de la Salud, Panamá, República de Panamá; 23 Center for Health Studies, Universidad del Valle de Guatemala, Guatemala City, Guatemala; 24 Núcleo de Medicina Tropical, Universidade de Brasília, Campus Universitário Darcy Ribeiro, Brasília, Brazil; 25 Centro Internacional de Entrenamiento e Investigaciones Médicas (CIDEIM), Cali, Colombia; 26 Fundación Nacional de Dermatología (FUNDERMA), Santa Cruz de la Sierra, Santa Cruz, Bolivia; 27 Department of Dermatology, Faculty of Medicine, Akdeniz University, Antalya, Turkey; 28 Department of Dermatology, Emam Reza Hospital, Mashhad, Iran; 29 Unit of HIV and Neglected Tropical Diseases, Institute of Tropical Medicine Antwerp, Antwerp, Belgium; 30 Instituto Medico la Floresta, Caracas, Venezuela; 31 Drugs for Neglected Diseases initiative (DNDi), Geneva, Switzerland; Ohio State University, UNITED STATES

## Abstract

**Introduction:**

Progress with the treatment of cutaneous leishmaniasis (CL) has been hampered by inconsistent methodologies used to assess treatment effects. A sizable number of trials conducted over the years has generated only weak evidence backing current treatment recommendations, as shown by systematic reviews on old-world and new-world CL (OWCL and NWCL).

**Materials and methods:**

Using a previously published guidance paper on CL treatment trial methodology as the reference, consensus was sought on key parameters including core eligibility and outcome measures, among OWCL (7 countries, 10 trial sites) and NWCL (7 countries, 11 trial sites) during two separate meetings.

**Results:**

Findings and level of consensus within and between OWCL and NWCL sites are presented and discussed. In addition, CL trial site characteristics and capacities are summarized.

**Conclusions:**

The consensus reached allows standardization of future clinical research across OWCL and NWCL sites. We encourage CL researchers to adopt and adapt as required the proposed parameters and outcomes in their future trials and provide feedback on their experience. The expertise afforded between the two sets of clinical sites provides the basis for a powerful consortium with potential for extensive, standardized assessment of interventions for CL and faster approval of candidate treatments.

## Author summary

The term ‘cutaneous leishmaniasis’ (CL) includes a range of manifestations affecting the skin caused by *Leishmania* parasites across several continents. While not life-threatening, CL can be invalidating and disfiguring, or become complicated. Today, there is no satisfactory treatment for CL that is effective and safe. Faced with no investments into developing drugs for CL, clinical researchers have tried many treatments over the years, but little progress has been made. One of the reasons is the lack of standardized methodologies in conducting these trials which makes it difficult to collate and compare results. Clinical researchers now realize that their efforts can be brought to fruition if common methodologies are available and applied. This paper summarizes the principles and parameters agreed upon by researchers of how to identify patients and how to measure treatment effects in a way that will make it possible to gather convincing evidence of whether a treatment works or not. Adhering to these principles will allow faster progress towards offering better care to patients with this neglected disease.

## Introduction

Cutaneous leishmaniasis (CL) is a disease caused by various *Leishmania* species affecting an estimated 0.7–1.2 million people each year in the Americas, the Mediterranean basin, the Middle East and Central Asia. In 2013, 95% of the cases reported to WHO occurred in 15 countries: Afghanistan, Algeria, Brazil, Colombia, Honduras, Iran (Islamic Republic of), Morocco, Nicaragua, Pakistan, Peru, Saudi Arabia, Syrian Arab Republic, Tunisia, Turkey, and Yemen [1,2]. In 2014, over 153 000 cases were reported to WHO from 10 high-burden countries [3,4].

Progress with the treatment of CL has been hampered by lack of investments in drug discovery and development, but also by the inconsistent methodologies that have been used to assess treatment effects [5]. This has resulted in significant scientific and financial waste, as a sizable number of trials conducted over the years have generated only weak evidence for treatment recommendations.

These weaknesses were exposed by two Cochrane systematic reviews on Old-World [6] and New-World [7] CL (OWCL and NWCL; the latter recently updated [8]). To correct these shortcomings, a series of steps were set in place towards achieving consensus on the main parameters that would help establish standardized, generally adoptable criteria in clinical investigations. This process started with a consultation jointly organized by the Special Programme for Research and Training in Tropical Diseases (WHO/TDR) and the World Health Organization Programme for Leishmaniasis at the Neglected Tropical Diseases Department (WHO/NTD) held in 2009, which led to a guidance paper in 2013 [9] that aimed to (i) provide clinical investigators with guidance for the design, conduct, analysis and report of clinical trials of treatments for CL, whilst recognizing the complexity of the disease; and (ii) enhance the capacity for high-quality trials that fulfil the requirements of International Conference on Harmonization (ICH) and Good Clinical Practice (GCP) standards.

A network of clinical trial sites for NWCL (RedeLeish [10]) was started with support by the Drugs for Neglected Diseases initiative (DNDi) in 2014, and discussions are underway to extend this network to OWCL (jointly with TDR).

## Methods

Basic parameters from the above-mentioned guidance paper were submitted to a group of OWCL and NWCL clinical trialists and discussed at workshops that took place in Tunisia (February 2016, hosted by the TDR regional training centre at Institute Pasteur, Tunis and organized by TDR) and in Brazil (June 2016, organized by DNDi). The meetings were attended by expert CL trialists representing 10 clinical study sites from 7 OWCL countries (Afghanistan, Burkina Faso, Ethiopia, Iran (Islamic Republic of), Morocco, Tunisia and Turkey) and 11 clinical study sites from 7 NWCL countries (Bolivia, Brazil, Colombia, Guatemala, Panama, Peru and Venezuela).

## Results

### Consensus on key methodological issues in clinical trials of CL treatments

**Fig 1:**
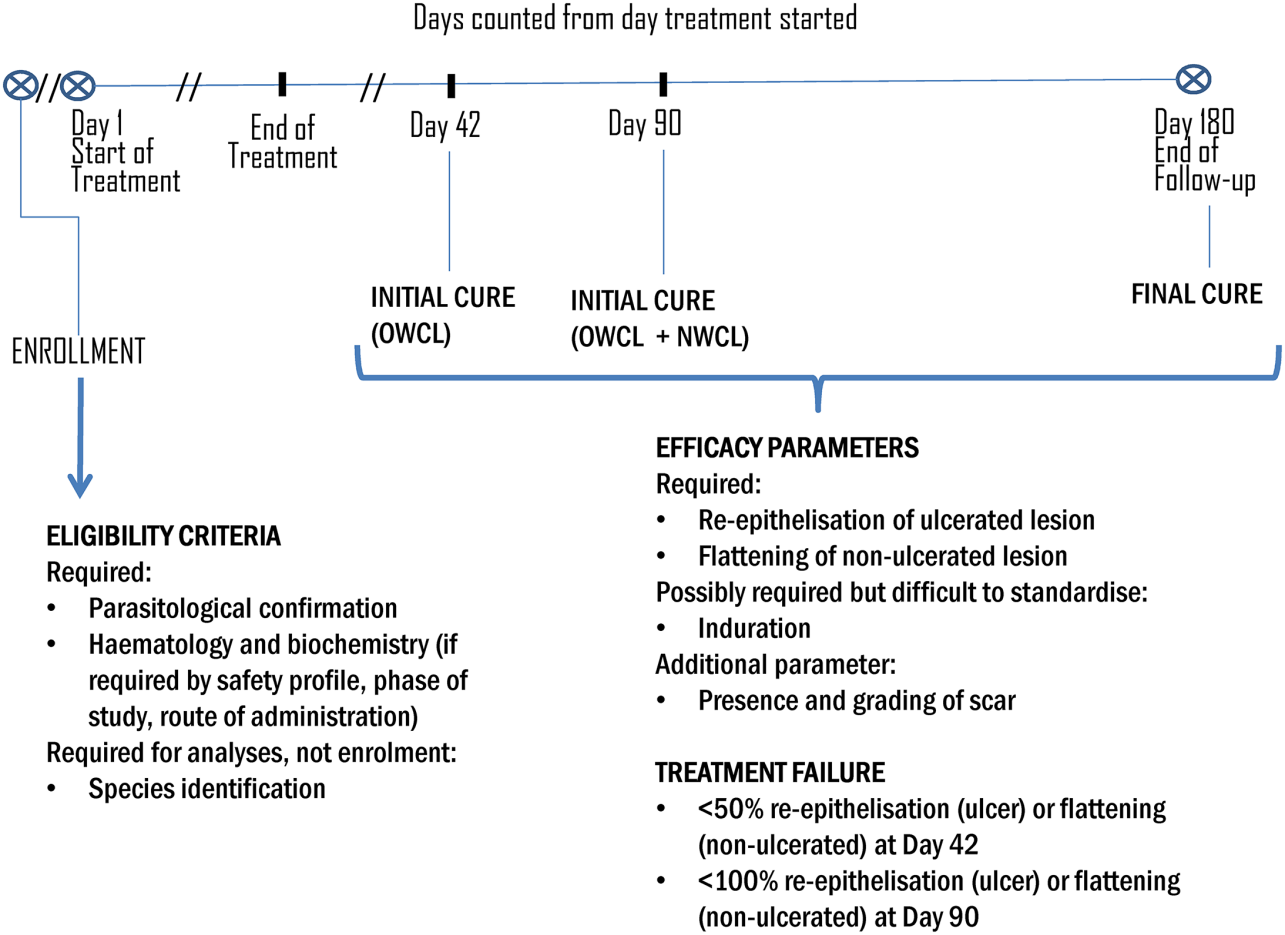
Days counted from day treatment started.

The degree of consensus and main issues are summarized in Table 1 for OWCL and NWCL, along with the revised parameters after the two above mentioned consultations. A diagrammatic representation can be found in Fig 1.

**Table 1:** Agreement on key parameters by OWCL and NWCL clinical researchers. ‘Standardized’ criteria are those as proposed in the reference paper Olliaro et al, 2013^9^; ‘updated’ criteria are those resulting from the consultation.

Key Parameters	Standardised (Olliaro et al, 2013)^9^	OWCL 10 sites, 7 countries		NWCL 11 sites, 7 countries		Updated
		Yes	No	Yes	No	
**Eligibility criteria**						
Only parasitologically-confirmed cases can be enrolled	Yes	100%		100%		Yes
*Leishmania* species identification required for enrolment	Yes/No	100%			100%	No
*Leishmania* species identification required for analysis	Yes/No	100%		100%		Yes
Baseline safety tests required (haematology, liver and renal function)*	Yes/No	100%		100%		Yes
**Efficacy parameters**						
Re-epithelization of ulcerated lesions	Yes	100%		100%		Yes
Flattening of non-ulcerated lesions	Yes	100%		100%		Yes
Induration	No	80%		91%		(Yes)
Redness	No	100%		100%		No
**Time at which initial cure should be assessed**						
End of treatment	No		100%		100%	No
Day 42	Yes	100%			100%	Yes OW; No NW
Day 90	Yes	100%		100%		Yes
**Time at which final cure should be assessed**						
Day 180 (6 months)	Yes	100%		100%		Yes
Day 360 (12 months)	Yes/No		100%		100%	No
**Follow-up counting from when?**						
From the end of treatment	No		100%		100%	No
From the beginning of treatment	Yes	100%		82%		(Yes)
**Definition of treatment failure**						
Day 42: <50% re-epithelization (ulcer) or flattening (non-ulcerated lesion)	Yes	100%		100%		Yes
Day 90: <100% re-epithelization (ulcer) or flattening (non-ulcerated lesion)	Yes	100%		100%		Yes
**Other efficacy parameters: stigma and cosmetic**						
Presence and grading of scar**	NA	100%		100%		Yes

* Depending on known side effect, safety profile, phase of development, drug class and route of administration

** Will require standardization

As for eligibility criteria, it was agreed that parasitological confirmation by visualization of the parasite (amastigotes in smears, promastigotes in culture) or molecular biology testing (primary PCR) is required for a patient to be enrolled in clinical trials; *Leishmania* species identification is not required for enrolment but is required for data analysis. The need for baseline safety tests (hematology, liver and renal function) depends on the risks associated with the treatment (route of administration and the phase of development), the drug’s chemical class, and the perceived or known liabilities of the treatment (expected toxicity).

As for the efficacy parameters, there was consensus about re-epithelization for ulcerated lesions and flattening for non-ulcerated lesions as primary efficacy measures. The majority of participants was for adding absence of induration as an efficacy parameter (though more difficult to standardize), while redness (inflammation) was thought to be not sufficiently reliable.

Even though the natural history and treatment response vary across the range of old and new world *Leishmania* species, it was agreed that initial cure should be assessed at Day 90–100 (Day 0 being the day of enrolment and Day 1 being the first day of treatment) since it provides the best chances to assess success or failure. In OWCL treatment trials, an additional earlier assessment at Day 42 should also be conducted and reported to capture the earlier clinical response observed in these species.

In addition, OWCL participants identified the need to document more clearly and quantify the rate of self-healing in *L*. *major*, in order to better inform decisions on follow-up and study design, such as the assessment of time-to-heal particularly after topical treatment, as a secondary outcome—which would require multiple assessments. NWCL participants discussed the need to collect evidence towards a future definition of “early failure” (before Day 42) based on the type of intervention/treatment being evaluated.

While it was acknowledged that, in some instances, treatment is provided until the lesion is considered cured (especially when evaluating topical or intralesional treatments), efforts should be made to report the number of cured subjects at day 42 and the initial cure at day 90.

Final cure should be assessed at Day 180 (6 months after initiating treatment); a 12-month follow-up was not deemed necessary. Nevertheless, NWCL participants identified the need to assess the ideal time of follow-up for final cure, and document the rate of late-responses and relapses between days 90–180 (3–6 months). This would provide important elements to understand the cost-effectiveness of a 6-month follow-up, and inform study design.

There was almost general agreement that time of follow-up is counted starting from the first day of treatment and not from the end of treatment. The main issue was how to deal with treatments of different duration. For instance, systemic antimonials are given for 14–30 days (see Tables 2 and 3); an initial assessment at day 42 counting from treatment start means 12–15 to 28–31 days after the end of treatment, compared to e.g. thermotherapy, which may be given in one single treatment.

**Table 2:** Site characteristics in Old World settings.

Country	Afghanistan	Burkina-Faso	Ethiopia	Ethiopia	Iran	Iran	Iran	Morocco	Tunisia	Turkey
**Institution**	National Malaria and Leishmaniasis Control Programme	Université Polytechnique de Bobo-Dioulasso	Armauer Hansen Research Institute (AHRI), Addis Ababa	University of Gondar, Gondar	Shiraz University of Medical Sciences, Shiraz	Tehran University of Medical Sciences	Emam Reza Hospital, Mashhad University of Medical Sciences	Department of parasitologic diseases, Ministry of Health, Rabat	Institut Pasteur, Tunis	Akdeniz University, Antalya & Ege University, Bornova, Izmir
**Area of work**	Kabul and Balkh	Bobo-Dioulasso, Ouagadougou	Addis Ababa, Silti, Ankober and Debretabor	Gondar, Northen part of Ethiopia	Shiraz and vicinity	Tehran	Mashhad	Taza, Sefrou, Errachidia, Ouarzazate,Azilal and Chichaoua	Sidi-Bouzid, Kairoan and Gafsa mainly (*L*. *major*), Tataouine *(L*. *tropica)*	Antalya and Adana
**Primary or referral center**	primary	both	referral	referral	both	both	both	both	both	primary
**Clinical research (GCP) experience**	no	yes	yes	yes	yes	yes	yes	no	yes	yes
**National treatment guidelines**	yes	yes	incomplete	yes	yes	yes	yes	yes	yes	yes
**N. of cases in country**	19589	532 in 2014 (investigator report)	342 (Investigators estimate 20,000–50,000)		21148			2555	3368 (investigators estimate up to 10,000)	3977
**Number of cases seen per year**	17,000–20,000	2013: 128; 2014: 144	~1500	~ 100 per site	1200–1500	250–500	300–400	100–400	200–600	50 in Antalya; 300 in Adana
**Leishmania species**	*L*. *tropica*	*L*. *major*	*L*. *aethiopica*	*L*. *aethiopica*	*L*. *major*, *L*. *tropica*	*L*. *major*, *L*. *tropica*	*L*. *major*, *L*. *tropica*	*L*. *major*, *L*. *tropica*	*L*. *major*, *L*. *tropica*	*L*. *major*, *L*. *tropica*, *L*. *infantum*
**Type of diagnosis, species identification**	direct smear	direct smear, PCR, biopsy	direct smear, PCR, culture	direct smear, PCR, culture species identification not done routinely	direct smear, PCR, biopsy	direct smear, culture, PCR for all patients	direct smear; selected cases: PCR and culture	direct smear, PCR	direct smear, PCR, culture	direct smear
**Age of subjects**	> 5 y.o.	adults	mainly 10–20 y.o.	teenagers and young adults	adults and children	adults and children	adults and children	adults and children	adults and children	adults and children
**Gender (F:M)**	50:50	50:50	50:50	50:50	50:50	35:65	50:50	50:50	50:50	50:50
**Emerging or stable foci**	stable	both)	both	stable	stable	stable	stable	both	both	stable
**Rural or (peri)urban setting**	both	both	both	rural	both	both	urban	both	both	rural
**Seasonality**	April-November	September-November	almost year-round	year-round	Peak: September-March	Peak: September-March	Peak: September-January	November-April	September-March	September-March
**Number of lesions**	>5	>5	~47% single	1–2 in 80%	few to many	mostly few, rarely multiple	mostly few, rarely multiple	few to many (1 to > 6)	1–2	1–2
**Type of lesion and size**	papule, nodule, ulcer; different sizes	mostly papule and ulcer; 20–40 mm also papulonodular, nodule	early lesions (<6 months) up to 80% nodular; chronic lesions (>6 months) >60% ulcerative	patch, ulcer, induration, plaque; ~50 mm	mostly ulcerated nodule or plaque; 15 mm	mostly ulcer	mostly papule and nodule; 20–40 mm	nodule, ulcer, plaque; < 40 mm	90% ulcer; 10–40 mm (mean 20 mm) also nodule and plaque	mostly papule and nodule; 10–20 mm
**Duration of lesion**	months to years	2–6 months	months to years	months to years	months to years	months to years	weeks to years, usually 3–6 months in daily practice	*L*. *major*: 2–6 months; *L*. *tropica*: mean 12 months	*L*. *major*: 1–6 months; *L*. *tropica*: mean 12 months	11 months
**Other manifestations**	lupoid, DCL		MCL, DCL	MCL, DCL	erysepeloid, sporotrichoid, lupoid	sporotrichoid	lupoid, recidivans, erysipeloid, sporothricoid, zosteriform, DCL	sporotrichoid, lupoid, erysepeloid	DCL	lupoid, chronic, erysepeloid, sporotrichoid
**Treatment (type/dose)**	IM antimonials 20mg/kg/d x 14-21d; IL injection based on size of lesion (2-4ml)	<5 lesions: IL antimonials, 2–3 ml/d x 2 days > 5 lesions: IM antimonials 20mg/kg/d x 21d, uo to 3 times	cryotherapy, IM antimonials 20mg/kg/d x 20d (max 850mg/day)	IM antimonials 20mg/kg/d x 30d, liposomal amphotericin B, paromomycin; oral miltefosine	cryotherapy one session per week, IM antimonials 20mg/kg/d x 15-20d; IL antimonials once a week	cryotherapy, heat therapy, IM antimonials 20 mg/kg/d x 14 days for *L*. *major*, 21 days for *L*. *tropica*; liposomal amphotericin B	cryotherapy, heat therapy, IM antimonials 20 mg/kg/d x 20-30d, IL antimonials x 1-2/week x 8–12 weeks, liposomal amphotericin B	IM antimonials 20mg/kg/21d IL antimonials 1-3ml x 2/week	IL antimonials, thermotherapy, cryotherapy	IM antimonials 20mg/kg/d x 20d, IL antimonials 1 ml/cm2 x 5–8 times, cryotherapy (monotherapy or combined with IL antimonials
**Duration of follow up**	1 month	until complete healing of lesions	3–6 months	3–6 months	3–6 months in routine clinical setting, until complete healing of lesions	until complete healing of lesions	3–6 months	until complete healing of lesions	1–6 months	12 months after end of treatment (every 3 months)

**Table 3:** Site characteristics in New World settings.

Country	Bolivia	Brazil	Brazil	Colombia	Colombia	Guatemala	Panama	Peru	Venezuela
**Institution**	Fundación Nacional de Dermatología, FUNDERMA. Santa Cruz de la Sierra	Serviço de Imunologia, Federal University of Bahia	Centro de Pesquisa René Rachou—FIOCRUZ, Belo Horizonte	Centro Internacional de Entrenamiento e Investigaciones Médicas (CIDEIM), Cali	Programa de Estudio y Control de Enfermedades Tropicales (PECET), Medellín	Center for Health Studies, Universidad del Valle de Guatemala	Instituto Conmemorativo Gorgas de Estudios de la Salud, Panamá	Hospital Cayetano Heredia, Universidad Peruana Cayetano Heredia, Lima	Instituto de Biomedicina Dr Jacinto Convit. Caracas Venezuela
**Area of work**	Santa Cruz and referred patients	Corte de Pedra, Tancredo Neves, Bahia	Minas Gerais	Mainly South-western	Caribbean coast, Amazon, Andean valleys, Pacific coast and eastern plains	El Peten and Alta Verapaz	Panama City and refereed patients	Andean and jungle areas	Metropolitan area
**Primary or referral center**	referral	both	referral	both	referral	referral	referral	referral	referral
**Clinical research (GCP) experience**	yes	yes	yes	yes	yes	yes	yes	yes	yes
**National treatment guidelines**	yes	yes	yes	yes	yes	yes	yes	yes	yes
**N. of cases in country/ (WHO official figures**	1683	19402		11433		254	1581	5888 (investigators estimate up to 8000)	1661
**Number of cases seen per year**	150–200	800–1,500	~90	~200	200	~100	~100	350–400	150
**Leishmania species**	*L*. *braziliensis*, *L*. *guyanensis*, *L*. *mexicana*	*L*.*braziliensis*	*L*.*braziliensis (95%)*	*L*. *panamensis*, *L*. *braziliensis*, *L*. *guyanensis*	*L*.*panamensis*, *L*.*braziliensis*	*L*. *braziliensis*, *L*. *mexicana*	*L*. *panamensis*, *L*. *guyanensis*, *L*. *braziliensis*	*L*. *braziliensis*, *L*. *peruviana*, *L*. *guyanensis*	*L*. *braziliensis and L*. *mexicana*
**Type of diagnosis, species identification**	direct smear; capability for culture and PCR	PCR	direct smear	direct smear, PCR, biopsy, monoclonal antibodies and isoenzymes	direct smear, PCR	direct smear, PCR	direct smear, culture, PCR, DTA	direct smear, PCR, culture; species identification	direct smear, culture, PCR, biopsy
**Age of subjects**	young adults	mainly adults	young adults	adults and children	adults and children	adults and children	adults and children	adults and children	adults
**Gender (F:M)**	10:90	30:70	30:70	20:80	50:50 (civilian population) 1:99 (military population)	45:55	33:67	50:50	38:62
**Emerging or stable foci**	stable	stable	stable	stable	stable	stable	stable	stable	stable
**Rural or (peri)urban setting**	both	rural	peri-urban	rural	both	rural	both	rural	both
**Seasonality**	all year-round	all year	all year-round	year-round	All year-round	all year-round	peak: March to July	peak: January-June	all year-round
**Number of lesions**	1–2	single	70% single	1 (1–3)	2	1–2	2–3	80% single	1–2
**Type of lesion and size**	ulcer; 25–30 mm	90% ulcer; 15 mm	70% ulcer; 80%<40 mm	80% ulcer; 90% <50 mm diameter	mainly ulcer (~80%); 20 mm	90% ulcers; 10–20 mm	90% ulcer; 10–20 mm	80% ulcer; 70% <30 mm	80% ulcer; 70% <30 mm
**Duration of lesion**	3–5 months in 90% cases	mean 1.5 month	~3 months	~2 months	2 months	3–4 months	3–4 weeks	mostly <3 months	1 month
**Other manifestations**	lymphangitis (35%), MCL (3–15%), DCL (5%)	MCL (3%), DCL(4%), atypical (3%)	lymphangitis (10–15%)	lymphangitis (18%), mucosal involvement (4%), disseminated (sporadic)	lymphangitis, MCL	lymphangitis (5%)	lymphangitis (10–20%)	lymphangitis (20–30%)—depending on time of disease	lymphangitis (<10%)
**Treatment (type/dose)**	IM antimonials 20 mg/kg/d x 20 d (85%); amphotericin B 0.5–1 mg (15%)	IM antimonials 20 mg/kg/d x 20 d	IL antimonials (60%); IM antimonials 20 mg/kg/d x 20 d (40%)	IM antimonials 20 mg/kg/d x 20 d. oral miltefosine (1.5–2.5 mg/kg/day); thermotherapy IL antimonials	IM antimonials: 20 mg/kg/day x 20d oral miltefosine 2.5 mg/kg/day x 28d pentamidine: 3–4 mg/kg/d x 3 doses every other day	IM antimonials 20 mg/kg/d x 20 d	IM antimonials 20 mg/kg/d x 20 d; amphotericin B for rescue treatment	IM antimonials 20 mg/kg/d x 20 d, amphotericin B rescue treatment	IM antimonials, no exact dose recommended; oral miltefosine
**Duration of follow up**	6 months	6 months	12 months	6 months	6 months	3–6 months	3–6 months	12 months	5 years

It was also agreed that treatment failure should be defined at Day 42 as less than 50% re-epithelialization (if an ulcer) or flattening (if a non-ulcerated lesion); and at Day 90 as less than 100% re-epithelialization or flattening, respectively.

It was suggested that the presence and grading of scars (cosmetic effects) should be assessed in a standardized way and included in future long-term treatment evaluation, as this represents an important parameter for patients because of the related stigma and social consequences. The results of qualitative studies using in-depth semi-structured CL patient interviews aimed at understanding the CL patient’s needs and expectations from treatment (paper in preparation) will also help inform study design.

### CL trial site characteristics and capacities

Site characteristics for OWCL and NWCL are summarized in Tables 2 and 3 respectively.

#### About the sites

‘Site’ here refers to a single or multiple treatment sites with different catchment areas covered by one group. Of the OWCL sites, 3 are referral centers, 2 primary and 5 both; for NWCL, 7 are referral and two both primary and referral. Capacity for good clinical practice (GCP) trials exists in 8/10 OWCL sites and 9/9 NWCL sites.

#### About the disease: OWCL

Species and diagnosis: Cases of both *L*. *major* and *L*. *tropica* cases are seen at 6/10 sites (one also *L*. *infantum*), while 2 sites have one species each and 2 have *L*. *aethiopica*. Parasitological diagnosis by direct smear is done at all sites. Capacity for polymerase–chain reaction (PCR) exists at 8 sites and culture at 5, though techniques are not always used routinely.

Age and Sex: All ages are affected in 7/10 sites. *L*. *aethiopica* affects mostly older children, adolescents and young adults. OWCL affects equally women and men, with more men being seen only in Iran.

Endemicity: Six out of 10 sites have stable transmission, while 4 see patients from both emerging and stable foci. There is no obvious pattern relating age and transmission. Burkina-Faso has a stable focus in Ougadougou and a newer one in Bobo-Dioulasso.

Setting: Patients seen at 6/10 sites are from both rural and periurban settings. In Burkina-Faso the Ougadougou focus is periurban, while the one in Bobo-Dioulasso is rural.

Seasonality: Cases are seen mostly in fall and winter at 7/10 sites with variable durations. Year-round transmission occurs in Ethiopia.

Number of lesions: Patients tend to present with few (1–2) lesions at 4 sites. Several lesions (>5) are seen in Afghanistan (*L*. *tropica*) and Burkina-Faso (*L*. *major*). The number of lesions varies in Iran and Morocco. There is no obvious pattern relating species and number of lesions.

Morphology and duration of lesions: Ulcerated, papular and nodular forms are seen across all sites. Lesions are up to 40 mm at 4 sites and up to 20 mm in 2 other sites. Other manifestations include disseminated (DCL, 5 sites), and others like lupoid, erysepeloid, sporotrichoid. *L*. *aethiopica* manifestations include DCL and mucocutaneous (MCL) forms.

Patients present with lesions that have lasted variably from weeks to years across the various sites. Tunisia and Morocco report that *L*. *major* patients seek treatment when lesions have been present for up to 6 months, *L*. *tropica*’s 12 months.

#### About the disease: NWCL

Species and Diagnosis: *L*. *braziliensis* is the most frequent species in 6/9 sites and present also in the other 3, where *L*. *panamensis* predominates. Other species found are *L*. *mexicana* and *L*. *guyanensis*. Parasitological diagnosis by direct smear is the technique used at all but one site in Brazil. Polymerase–chain reaction (PCR) exists at 8 sites and culture at 4 but these are not always done routinely.

Age and Sex: All ages are affected in 5/9 sites. Men represent approximately two-thirds of patients at 5 sites and 90% at another one, while the other 3 sites have almost equal representation of women and men.

Endemicity: All sites have stable transmission

Setting: Patients seen at 4/9 sites are from both rural and periurban settings, 4 rural and one periurban.

Seasonality: Cases are seen all year-round in all sites, some with seasonal peaks.

Number of lesions: Patients tend to present with single or few (up to 3) lesions.

Morphology and duration of lesions: Ulcerated lesions predominate at all sites. Other manifestations include lymphangitis (5–35% at 8/9 sites), MCL (3 sites) and DCL (2 sites).

Patients present with lesions that have lasted from 3 weeks to 5 months, but mostly not exceeding 3 months (7 sites). Bolivia reports seeing increasing numbers of chronic cases with disease lasting for over 18 months.

#### About treatment and follow-up: OWCL

Treatment: Intramuscular (IM) antimonials at 20 mg/kg/day for 14–30 days is used at 8/9 sites. Intralesional (IL) antimonials are also used at 7 sites at variable dosages (volume injected, number of doses, and duration of treatment). Choice of treatment may depend on the number of lesions (IL if less than 5 lesions, otherwise IM in Burkina-Faso) or species (IM antimonials are given daily for 14 days in case of *L*. *major* and 21 if *L*. *tropica* at one site in Iran). In addition, local antiseptics are regularly applied at 2 sites. Cryotherapy and/or thermotherapy are also used at 5 sites (alone or combined with IL injections). Other medications available are liposomal amphotericin B (2 sites in Iran), paromomycin and oral miltefosine at one site in Ethiopia.

Duration of follow-up: Practice varies greatly; 3 sites follow patients up routinely until complete healing of lesions; others follow patients up for a fixed duration from 1 month to 12 months (3–6 months in 4 sites).

#### About treatment and follow-up: NWCL

Treatment: Intramuscular (IM) antimonial at 20 mg/kg/day for 20 days is used at all sites. Second-line treatment consists of amphotericin B deoxycholate at 3 sites, oral miltefosine (2 sites), or pentamidine (1 site). Intra-lesional antimonials are used only in one site in Colombia and one in Brazil.

Duration of follow-up: Six (6) sites follow patients up for 6 months, 2 for 12 months, and one for 5 years.

## Discussion

The consensus reached among participants during the two meetings allows standardization of future clinical research across OWCL and NWCL sites—a major issue which has hampered our collective ability to generate strong evidence for treatment guidelines and policy. We encourage CL researchers to adopt and adapt if so required the proposed parameters and outcomes in their future trials. Furthermore, the expertise afforded between the two sets of clinical sites provides the basis for a powerful consortium with potential for extensive, standardized assessment of interventions for CL.
